# Purification and characterization of an extracellular laccase from solid-state culture of *Pleurotus ostreatus* HP-1

**DOI:** 10.1007/s13205-013-0129-1

**Published:** 2013-03-16

**Authors:** Hardik Patel, Shilpa Gupte, Mayur Gahlout, Akshaya Gupte

**Affiliations:** Department of Microbiology, N. V. Patel College of Pure and Applied Sciences, Vallabh Vidyanagar, 388 120 Gujarat India

**Keywords:** Laccase, *Pleurotus ostreatus*, Purification, N-terminal sequencing, ABTS

## Abstract

A native isolate of *Pleurotus ostreatus* HP-1 (Genbank Accession No. EU420068) was found to have an excellent laccase producing ability. The extracellular laccase was purified to electrophoretic homogeneity from copper sulphate induced solid-state fermentation medium by ammonium sulphate precipitation and ion-exchange chromatography. The enzyme was determined to be monomeric protein with an apparent molecular mass of 68,420 kDa, and an isoelectric point (p*I*) of 3.5. The inductively coupled plasma spectroscopy showed a presence of iron, zinc and copper in the purified enzyme. The absorption spectrum in the range of 200–700 nm showed the maximum absorption at 610 nm characteristic of fungal laccase and corresponding to the presence of type I copper atom. The laccase was stable at different temperatures up to 70 °C and retained 61 % activity at 50 °C. The enzyme reaction was inhibited by cysteine; sodium azide and EDTA. The enzyme oxidized various known laccase substrates, its lowest *K*_m_ value being for *ortho*-dianisidine and highest *K*_cat_ and *K*_cat_/*K*_m_ for ABTS. The purified laccase exhibited different pH optima for different substrates. The N-terminal sequence did not show any similarity with N-terminal sequence of other species of genera *Pleurotus*.

## Introduction

Laccases (benzenediol: oxygen oxidoreductase, E.C. 1.10.3.2) are multinuclear copper-containing enzymes that catalyse the oxidation of a variety of phenolic and inorganic compounds, with the concomitant reduction of oxygen to water. Laccases are widely distributed in nature and have also been detected in plants, insects, bacteria and especially in fungi (Majeau et al. 2010). These oxidative enzymes are particularly abundant in white-rot Basidiomycete fungi, which are capable of degrading lignin in vivo. Fungal laccase are known to catalyze the polymerization, depolymerization and methylation and/or demethylation of phenolic compounds (Leonowicz et al. 1985). Laccases are either monomeric or multimeric copper containing glycoprotein’s, which may exhibit additional heterogeneity because of variable carbohydrate content or difference in copper content or because they are expressed as the products of multiple genes (Dahiya and Singh 1998). The implications of laccases in biotechnology follow from the foregoing characteristics from their already demonstrated effectiveness as agents for selected bioremediation, catalysts for regiospecific biotransformations, participants in biosensor constructs, and from their considerable retention of activity in organic solvents (Slomczynski et al. 1995). Fungal laccases have ability in the degradation of toxic fungal metabolite, such as aflatoxin B1, in ethanol production, manufacturing of cream and wine clarification. These characteristics have led to laccases being qualified as “eco-friendly” enzymes (Alberts et al. 2009; Lu et al. 2007).

The purification of a laccase is an essential step for the determination of accurate kinetic parameters due to the possible presence of compounds from the host fungus that may act as natural mediators, or the presence of similar enzymes that may exhibit significantly different reaction kinetics (Rittstieg et al. 2003). The most commonly used method for laccase purification is salt elution from an anion-exchange resin, probably due to the higher stability of laccase at neutral to slightly alkaline pH, as well as the isoelectric point (*pI*) of laccases.

In the present work, a systematic study was conducted to purify and characterizes the laccase produced by *Pleurotus ostreatus* HP-1. Properties investigated in this study included molecular mass, isoelectric point, the effect of pH, temperature, inhibitor on enzyme activity, thermal stability and substrate specificity. The appropriate knowledge about structural and functional properties of *P. ostreatus* HP-1 laccases will further help in the elucidation of physiological function of this enzyme.

## Materials and methods

### Chemicals

The 2,2′-azino-bis-3-ethyl-benzthiazoline-6-sulphonic acid (ABTS) and Guaiacol were purchased from Sigma Chemical (St. Louis, USA). The 2,6-dimethoxyphenol was purchased from Lancaster (Lancs, UK). The silver nitrate, sodium carbonate, acrylamide, bis-acrylamide, acetone, formaldehyde were procured from Qualigens (India). All other chemicals were used of analytical grade and of highest purity available.

### Ammonium sulphate precipitation of laccase

For the production of laccase from *P. ostreatus* HP 1 (Genbank Accession No. EU420068) optimum culture conditions were used as described in our previous study (Patel et al. 2009). At the end of fermentation cycle the content from the flasks were squeezed through muslin cloth and the filtrate obtained was centrifuged at 10,000 rpm for 10 min at 4 °C. The supernatant thus obtained was subjected to the total protein precipitation with ammonium sulphate in the range of 0–75 % saturation. Dialysis was performed against 100 mM sodium acetate (pH 4.5).

### Purification of laccase by column chromatography

The purification was carried out with the help of AKTA purifier, Amersham Biosciences (USA). The dialyzed sample was applied to a DEAE-Sepharose column (1 × 15 cm) previously equilibrated with 50 mM Na-phosphate buffer (pH 6.0) (Buffer-A). Other mobile phase was 50 mM Na-phosphate buffer with 1 M NaCl (pH 6.0) (Buffer-B). The protein samples (5 ml) were eluted with a gradient elution system in three steps: Step 1: 80 % of Buffer A and 20 % of Buffer B was run for 75 min. Step 2: 20 % of Buffer A and 80 % of Buffer B was run for 80 min. Step 3: 100 % of the Buffer B was run for 60 min. Re-equilibration of column after purification was carried out with 100 % of Buffer A (Xiao et al. 2004).

### Enzyme activity

#### Laccase

Laccase activity (E.C. 1.10.3.2) was determined by measuring the oxidation of ABTS. Increase in absorbance for 3 min was measured spectrophotometrically (Elico BL-198, Hyderabad, India) at 420 nm (Niku et al. 1990). The reaction mixture contained 100 μl of 50 mM ABTS and 800 μl of 20 mM Na-acetate buffer (pH 4.5) and 100 μl of appropriately diluted enzyme extract. One unit of enzyme activity was defined as amount of enzyme that oxidized 1 μM of substrate per min at room temperature.

### Enzyme characterization

#### Molecular weight determination

The dialyzed sample of laccase was subjected to denaturing and non-denaturing PAGE on 12 % gel. SDS-PAGE was performed to determine sample purity and approximate molecular mass of laccase. The approximate molecular mass of the laccase was determined by calibration against broad range molecular weight markers (BioRad), which contained the proteins phosphorylase B (97.4 kDa), bovine serum albumin (66 kDa), ovalbumin (43 kDa), carbonic anhydrase (29 kDa), soybean trypsin inhibitor (20.1 kDa), and lysozyme (14.3 kDa). Non-denaturing PAGE was performed to ascertain which protein correlated to laccase activity. To ascertain accurate molecular weight of purified laccase matrix-assisted laser desorption/ionization-time of flight (MALDI-TOF) was performed.

#### Activity staining

Activity staining for laccase was performed with ABTS as the substrate. Non-denaturing (Native-PAGE) gels were allowed to stand in 100 ml of sodium acetate buffer (100 mM, pH 4.5) with 1 ml of 10 mM ABTS for 20–30 min. Laccase activity spots were indicated by the development of a green colored band.

#### Determination of p*I*

Analytical isoelectric focusing on polyacrylamide gel (IEF-PAGE) in the range 2.5–7.0 was performed on a Mini-IEF apparatus (Biorad, Richmond USA). The pH gradient was measured by using the following standards: human carbonic anhydrase (p*I* = 6.55), bovine carbonic anhydrase (p*I* = 5.88), β-glucosidase (p*I* = 5.20), soybean trypsin inhibitor (p*I* = 4.50), glucose oxidase (p*I* = 4.15), amyloglucosidase (p*I* = 3.50) and pepsinogen (p*I* = 2.80).

#### N-terminal sequencing and MALDI-TOF analysis

The N-terminal sequencing was performed on Applied Biosystems (Precise Protein Sequencer Company) and matrix-assisted laser desorption/ionization-time of flight (MALDI-TOF) using Axima-CFR, Kartos Analytical (A Shimadzu Group Company) at IIT, Powai (Mumbai, India).

#### Effect of different pH

The pH optima was determined over a range of pH 2–9. The pH optima of an enzyme was performed by using three different buffer systems comprising of: 100 mM sodium acetate buffer (pH 2.0–5.0); 100 mM sodium phosphate buffer (pH 6.0–8.0) and 100 mM Tris–HCl buffer (pH 8.0–9.0).

#### Optimum temperature and thermal stability

The effect of temperature on laccase activity was determined by oxidation of ABTS for 3 min at temperature ranging from 20–80 °C with an interval of 10 °C. The thermal stability was determined under same assay conditions in a temperature range of 20–80 °C. Sodium acetate buffer (pH 4.5, 100 mM) was used for all the reactions, at optimum pH of laccase.

#### Enzyme kinetics

Four different substrates (ABTS, Guaiacol; DMP and *O*-dianisidine) that could be oxidized by laccase were used in varying concentrations (20–100 mM). Apparent *K*_m_ was determined by Lineweaver–Burk plot for laccase with various substrates used.

#### Inhibition studies

Three different potential inhibitors sodium azide (NaN_3_) cysteine and ethylene diamine tetra acetic acid (EDTA) were evaluated to test the inhibition properties on laccase.

#### Metal content of laccase

The purified laccase was checked for presence of different metal ions such as copper, iron and zinc by inductively coupled plasma spectroscopy–optical emission spectroscopy (ICP–OES) (Perkin Elmer, USA, Optima-3300 RL).

#### Absorption spectrum of laccase

The UV–visible spectrum of the purified laccase was determined at wavelengths between 200 and 700 nm at room temperature in 100 mM acetate buffer (pH 4.5) using a UV–visible spectrophotometer (ELICO, BL-198, Hyderabad, India).

## Results and discussion

### Purification

Optimum culture conditions were used for the production of laccase from *P. ostreatus* HP 1 (Genbank Accession No. EU420068) as described in our earlier study (Patel et al. 2009). The crude filtrate obtained was then subjected to the purification process. The complete precipitation of laccase from the crude filtrate required at least 75 % saturation of the supernatant with ammonium sulphate. After the application of dialysis further purification process was carried out with DEAE-sepharose column (1 × 15 cm), using gradient elution in three different steps, each comprising combination of two different buffers. Before the first step, the flow through of the column was observed for the first 60 min, which is the removal of the unbounded material from the column. No laccase activity was observed in any of the fractions collected in the first step. A sharp protein peak with laccase activity was obtained in second step, Maximum recovery of laccase occurred in this step. Negligible loss of laccase activity (0.031 U ml^−1^) was observed in the 3rd step and was therefore not taken into any consideration. At last, the purified enzyme obtained was subject to freeze drying. This strategy resulted in 13.13-fold purification with 77.63 % yield of laccase enzyme. Sahay et al. (2008) reported 10.71-fold purification with 3.46 % yield from *Pleurotus sajor*-*caju* MTCC 141. Diaz et al. (2010) reported 32.7 and 31.2 purification fold with 2.2 and 2.6 % yield for Lac I and Lac II isoenzymes from *Coriolopsis rigida*. Comparing to other studies, the results obtained were quite encouraging. However, Junghanns et al. (2009) reported 117-fold purification of laccase enzyme with very low yield of 0.95 %.

### Characterization of purified laccase

**Fig. 1:**
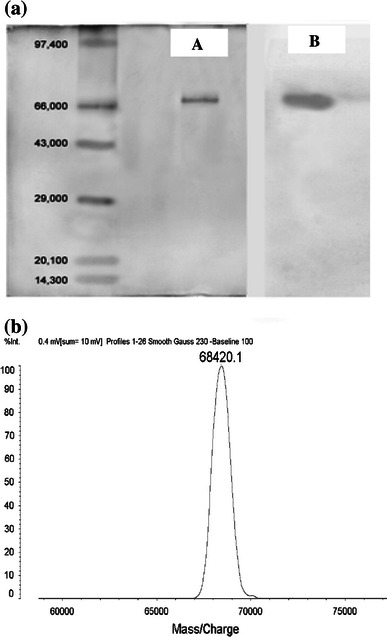
**a** The SDS-PAGE (*A*) indicating single band of protein with molecular weight of approximately 66,000 Da which corresponds to the single band showing *blue color* of laccase activity in activity staining (*B*). **b** The *peak* of MALDI-TOF indicating the actual molecular weight of the purified laccase

The purified laccase was further analyzed and characterized by native-PAGE, SDS-PAGE, MALDI-TOF, IEF, ICP-OES, N-terminal sequencing and UV–visible spectrum analysis. The non-denaturing gel (native-PAGE) with duplicate sets of samples was bisected and half was stained with silver nitrate, and the other half was stained with ABTS to determine which band correlated to laccase activity (Fig. 1a). Purified laccase from *P. ostreatus* HP-1 was shown to be homogeneous with single protein band according to the size with SDS-PAGE (Fig. 1a) as well as with native-PAGE (Fig. 1a). The relative molecular mass of the laccase protein was determined, and was found to be more than 68,000 Da, and it was further confirmed with MALDI-TOF analysis which revealed that the protein had a molecular weight of 68,420 Da (Fig. 1b). Zymogram analyses were performed for both, the denaturing as well as non-denaturing electrophoresis gels. The activity staining of laccase, with ABTS as substrate, revealed that the single protein band corresponded with activity of the laccase. The relative molecular mass of *P. ostreatus* HP-1 laccase compares well with the 68,000 Da laccase of *P. sanguineus* reported by Gonzalez et al. (2008). The relative molecular mass of laccase purified from *Lentinulaedodes* and *Phoma* sp. reported to be 58.5 and 75.6 kDa, respectively (Junghanns et al. 2009; Ulrich et al. 2005).

The isoelectric point p*I* of purified laccase was found to be 3.5 which is more acidic; however, p*I* values can vary widely, from 2.9 to 6.9 (Baldrian 2006). The concentrated enzyme solution was slight yellow brown in color. Certain laccases have been found to be yellow or yellow brown rather than the blue color that is expected for laccases. Yellow laccases and blue laccases from the same organism had similar copper contents. It was proposed that yellow laccases, under normal aerobic conditions, did not maintain their copper centers in the oxidised state of resting enzymes. The binding of low molecular mass phenolic material from lignin degradation could contribute to such a change of enzymatic property (Leontievsky et al. 1997).

### Presence of metal ions in purified laccase

It has been also found that laccases that do not have the blue color, can have copper, zinc and iron atoms instead of the classical four copper atoms. The ICP analysis of enzyme sample revealed the presence of two other metals, iron and zinc. The ratio for the metal ions detected by ICP was found to be 1:1:2 stoichiometry of copper/iron/zinc. Similar results were reported for the laccase isoenzyme POXA1 obtained from *P. ostreatus* (Palmieri et al. 1993).

### N-terminal sequence analysis

The terminal amino acid sequence of laccase from *P. ostreatus* HP-1 was when compared with other *Pleurotus* spp. (Table 2). It has been found that the N-terminal sequence of purified laccase did not show any similarity with those of the laccases from other *Pleurotus* spp. This may be due to truncated amino acids obtained during sequencing. However, from the present investigation it is difficult to draw a conclusion on such dissimilarity of N-terminal sequence of laccase from *P. ostreatus* HP-1 with other *Pleurotus* spp.

### Spectrophotometric analysis of purified laccase

The absorption spectrum of laccase in the range 200–700 nm was also performed. An absorption maximum at 610 nm shows the characteristic of fungal laccase copper atom type I. Which compares well with laccase enzyme from *Paraconiothyrium variabile* showed a peak around 600 nm, indicating the presence of copper atom type I (Forootanfar et al. 2011). The type 3 binuclear copper was absent in laccase of *P. ostreatus* HP-1 as there was no shoulder at 330 nm. The type 3 binuclear copper in present in both the laccases of *Pleurotus eryngii* (Munoz et al. 1997). This combination of molecular features (unusual color and absence of the in the absorption spectrum) were found previously in ‘yellow’ laccases of the fungi *Panus tigrinus*, *Phlebia radiata*, and *Phlebia tremellosa* (Leontievsky et al. 1997). This was again an evidence of this laccase as ‘yellow’ other than the typical ‘blue’ laccase found in many *Pleurotus* spp.

### Kinetic properties of laccase

Values of *K*_m_ and *K*_cat_ of different laccases widely vary for the same substrate. The majority of laccasses combine a high affinity for ABTS and syringaldazine with a high catalytic constants, where as guaiacol and 2,6-dimethoxyphenol are oxidized markedly slower and the corresponding Michaelis constants are significantly higher (Table 1). The *K*_m_ values (Table 1) of laccase from *P. ostreatus* HP-1 towards the various substrates indicated that the binding affinity towards the different substrates were in the order: *O*-dianisidine > ABTS > Guaiacol > DMP. In contrast the affinity (*K*_m_ value) of the *Myrioconium* sp. laccase towards typical laccase substrates follows the rank order syringaldazine > DMP > ABTS, where DMP is more specific substrate than ABTS for laccase (Martin et al. 2007). The values reported for the parameter, *K*_cat_, is a representation of the rate the catalytic process (Table 2).

**Table 1:** A comparison of kinetic properties of purified laccase from *Pleurotus ostreatus* HP-1 with other reported *Pleurotus* spp.

Source of laccase	Substrate	*K*_m_ (mM)	*K*_cat_ (s^−1^)	*K*_cat_*/K*_m_ (mM^−1^ s^−1^)	References
*Pleurotus ostreatus* HP-1	ABTS	46.51	244.32	5.25	Present study
*Pleurotus ostreatus* HP-1	DMP	400	208.33	0.52	Present study
*Pleurotus ostreatus* HP-1	Guaiacol	100	208.33	2.08	Present study
*Pleurotus ostreatus* HP-1	*O*-dianisidine	23.52	43.85	1.86	Present study
*Pleurotus ostreatus POXA1*	ABTS	0.09	5,833	64,815	Palmieri et al. (1993)
*Pleurotus ostreatus POXC*	Guaiacol	1.2	2.5	2.1	Palmieri et al. (1993)
*Pleurotus pulmunarius* Lcc2	ABTS	0.210	1,520	7,238	De-Souza and Peralta (2003)
*Pleurotus pulmunarius* Lcc2	Guaiacol	0.550	310	563	De-Souza and Peralta (2003)
*Pleurotus pulmunarius* Lcc2	Syringaldazine	0.012	654	54,500	De-Souza and Peralta (2003)
*Pleurotus florida*	*O*-dianisidine	0.45	1.86 × 10^6^	4.13 × 10^7^	Das et al. (2001)
*Pleurotus florida*	Guaiacol	2.81	9.3 × 10^5^	3.31 × 10^5^	Das et al. (2001)

*K*_m_ = Michaelis constant, *K*_cat_ = turnover number, *K*_cat_/*K*_m_ = measures of catalytic efficiency

**Table 2:** Comparison of N-terminal amino acid sequence of laccase from *Pleurotus ostreatus* HP-1 with other *Pleurotus* spp.

Organism	N-Terminus amino acid sequence	References
*Pleurotusostreatus* HP-1	S L W V A L W M S A – – –	Present study
*Pleurotus florida*	– – – P A G N M Y I V N E	Das et al. (2001)
*Pleurotus ostreatus*	A I G P A G N M Y I V N E	Palmieri et al. (1993)
*Pleurotus eryngii*		
L1	A – K K L – D F H I I N N	Munoz et al. (1997)
L2	A T K K L – D F H I I N N	
*Pleurotus ostreatus* POX1	S I G P N G T L N I A N K	Palmieri et al. (1993)

The ratio *K*_cat_/*K*_m_ gives an indication of each enzyme and therefore allows us to compare the efficiencies of the different enzymes. Table 1 shows that ABTS is most efficiently oxidized by laccase as it exhibits the highest values for *K*_cat_/*K*_m_ ratio for all the substrates studied. The guaiacol was the second efficient substrate oxidized by the laccase but it was less reactive towards *O*-dianisidine and DMP. The overall order of efficiency of enzyme for different substrates studied can be given as ABTS > guaiacol > *O*-dianisidine > DMP. The *K*_cat_ value for ABTS (244.32 s^−1^) was lower than those found for several other white-rot fungi laccases, such as those of *Trametesmulticolor* (510 s^−1^), *Coriolus hirsutus* (260 s^−1^) but higher than those reported *Picnoporus cinnabarinus* (920 min^−1^) and *Phellinus ribis* (8.0 × 10^4^ min^−1^) (Leitner et al. 2002; Shin and Lee 2000; Min et al. 2001).

### Inhibition study

The activity of laccases is inhibited by various organic and inorganic compounds (Morozova et al. 2007). Anions such as the halides, azide, cyanide and hydroxide bind to the types 2 and 3 copper atoms of laccases, which disrupts the electron transfer system, resulting in enzyme inhibition (Gianfreda et al. 1999). Three potential inhibitors (Sodium azide, EDTA and cysteine) were evaluated to check the inhibition properties of laccase. The EDTA and cysteine inhibited the laccase to a lesser extent. The results shows the different inhibition constants (*K*_i_) and type of inhibition observed in this study. The sodium azide is an inhibitor of metallo-enzymes showed ‘non-competitive’ inhibition which was classified as same *K*_m_ for both i.e. with inhibitor and without inhibitor and decrease in *V*_max_. This observation was not consistent with the findings of Heinzkill et al. (1998) who noted competitive inhibition with sodium azide for laccase from four different species of fungi, and average *K*_i_ of approximately 1 μM. The *K*_i_ obtained for the *P. ostreatus* HP-1 was in the region of 0.0165 mM, indicating that the presence of the sodium azide is less inhibitory to laccase. The cysteine showed ‘competitive non-competitive or mixed inhibition’ because the pattern lies between competitive and non-competitive inhibition of laccase, where a series of line intersecting between*y* axis intercept and*x* axis intercept in Lineweaver–Burk Plot. Although EDTA exhibited the uncompetitive type of inhibition classified as decrease in both *K*_m_ and *V*_max_. In the present study, the oxidase inhibitor sodium azide caused complete inhibition of laccase at concentration of 0.05 mM. The study by Iyer and Chattoo (2003) reported the laccase from rice blast fungus *Mannaporthe grisea* was inhibited up to 99 % at 1 mM concentration of sodium azide. The possible reason of laccase enzyme inhibition is the binding of NaN_3_ to the metal ion sites of laccase, that affect internal electron transfer, which ultimately affect the overall oxidation process catalyzed by laccase (Ryan et al. 2003). Chelating agent EDTA inhibited the enzyme only at higher concentrations which was in accordance with the earlier study by De-Souza and Peralta (2003). The EDTA was not an efficient inhibitor of laccase, which was in line with the earlier studies by other researchers (Heinzkill et al. 1998; Liu et al. 2010).

### Temperature stability

**Fig. 2:**
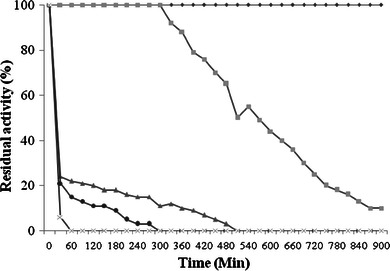
Effect of different temperature 30 °C *wide diamond*, 40 °C *square*, 50 °C *triangle*, 60 °C *diamond* and 70 °C *cross* on stability of laccase

Temperature stability of laccase was studied at various temperatures for various time periods in 100 mM acetate buffer pH 4.5. The enzyme was quite stable at −4 °C for months. When laccase was incubated at 30 °C, it was stable up to 18 h (Fig. 2). Purified laccase was quite stable at 40 °C for 5 h (Fig. 2). However, at 50 °C laccase was found stable for 10 min and considerable loss of activity was observed after 12 min (Fig. 2). The brief increase in the activity of the *P. ostreatus* HP-1 laccase after 10 min at 50 °C was probably due to the unfolding of the protein structure (Ryan et al. 2003). In the initial 15 min of incubation the half of the laccase activity was found to be lost at 60 °C (Fig. 2), whereas rapid inactivation of the enzyme was observed at 70 °C (Fig. 2). Comparable results of thermostability was also reported by *Agaricus blazei* laccase (Ulrich et al. 2005) and *Cerrena unicolor* LacC2 (Lisova et al. 2010). The laccase from *P. ostreatus* HP-1 showed its optimum activity at 50 °C which was quite similar to *P. ostreatus* (Palmieri et al. 1993) and L_2_ laccase enzyme from *Pleurotusflorida* (Das et al. 2001). Laccase from *P. ostreatus* was almost fully active in the temperature range of 40–60 °C and showed half life of 30 min at 60 °C (Das et al. 2001). In the present study, the laccase was active in the temperature range of 40–70 °C with half life of 15 min at 60 °C. Laccase was almost completely inactivated after 15 min at 70 °C. Munoz et al. (1997) showed that the laccase I and II of *Pleurotus eryngii* retained 3 and 10 % residual activity, respectively, at 60 °C after 30 min. Gonzalez et al. (2008) reported 3.7 % residual activity of *Pycnoporous sanguineus* laccase after incubation at 70 °C for 1 h. In contrast Tong et al. (2007) reported *Trametes* sp. LacE with half life of 1.3 h at 60 °C. The differences in the effect of different temperatures on laccase activity might be related to, some extent, to the number of disulphide bonds in the molecule. In addition, the thermal dissociation might also participate in the temperature profile (Xu et al. 1996).

### Effect of different pH

The optimum pH for the majority of fungal lacasses found in the range of 3.0–4.0 when organic donors of hydrogen atoms are used as substrates, and the pH dependence curve is bell-shaped (Stoilova et al. 2010) and such profile of laccase activity on oxidation of phenolic compounds is caused by two effects. On one hand, with increase in the pH of the solution, ionization potential of phenolic compounds decreases with as a result of formation of phenolate anion that has to enhance the rate of the enzymatic reaction. On the other hand, with increase in the pH of the solution the rate of laccase catalyzed reactions decreases at the cost of −OH binding with the T2/T3 site of the enzyme.

**Fig. 3:**
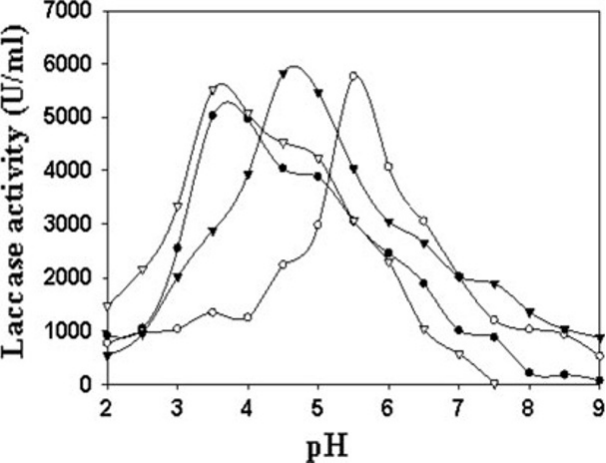
Effect of different pH of the buffer on oxidation of different substrates by purified laccase. *filled circle*, *open circle*—dianisidine (U ml^−1^), *open circle* guaiacol (U ml^−1^), *inverted filled triangle* ABTS (U ml^−1^), *inverted open triangle* DMP (U ml^−1^)

The purified enzyme was tested for its characteristics under different pH conditions. The pH optima of purified laccase on different substrates were determined over a range of pH starting from 2.0 to 9.0. The pH profile of *P. ostreatus* HP-1 laccase showed maximum activity at pH 4.5 when ABTS was used as a substrate (Fig. 3). *Pycnoporus cinnabarinus* laccase showed to have optimum pH of 4 for ABTS (Eggert et al. 1996). *P. ostreatus* HP1 laccase exhibited maximum activity at 3.5 pH for both DMP and *O*-dianisidine (Fig. 3). *Cerrena unicolor* VKMF-3196 LacC2 demonstrated maximum activity at pH 3.8 for DMP (Lisova et al. 2010), which is quite comparable with our results. Whereas for guaiacol pH 5.5 was found to be optimum for laccase activity (Fig. 3). Laccase from *Phoma* sp. UHH 5-1-03 showed optimum pH 5.0 when guaiacol was used as a substrate (Junghanns et al. 2009). A gradual decrease in the oxidation rate of various substrates was observed at higher pH, which may be due to ionization of critical amino acids (Asp and Glu), indicating that the enzyme was inactive at higher pH (Salony et al. 2006). The highest activity of *P. ostreatus* laccases with respect to pH profile also varied with the changes of substrates (Palmieri et al. 1993). The pH optima for activity depends on the substrate selected. For example, *P. ostreatus* has a pH optimum range of 3–3.5 for the oxidation of ABTS, but when the pH optima is determined for guaiacol and syringaldazine it is 5.6 and 6.7, respectively (Palmieri et al. 1993). This can be attributed to the reaction mechanism of the laccase. Whether the mechanism involves abstraction of an electron or hydrogen. The latter will be affected by changes in pH. As well as, the reaction depends on the redox potential of the substrate. The pH affects the ionization state of the substrate and therefore affects its ability to act as a reducing substrate (Majcherczyk et al. 1999).

## Conclusion

The laccase from *P. ostreatus* HP-1 was purified to apparent electrophoretic homogeneity with high protein recovery. The improved catalytic properties of laccase, such as the high affinity for its substrate and relatively high turnover numbers, especially for the oxidation of ABTS, indicate that this enzyme may prove to be highly competitive for industrial application. The specificity constant (*k*_cat_/*K*_m_), which takes into consideration both the specificity of the laccase for its substrate (*K*_m_) as well as the catalytic efficiency (*k*_cat_), was different to the specificity constant of “white” laccase from *P. ostreatus*. Owing to these improved catalytic properties, the broad pH range for the oxidation of ABTS, and production of this laccase in sufficient yields may provide a valuable alternative enzyme source for industrial applications. The sequence information obtained from purified laccase produced by *P. ostreatus* HP-1 did not showed any similarity to that of laccase from other *Pleurotus* spp. However, such kind of dissimilarity of N-terminal sequence could not be explained at this time. If combination made between the data obtained from visual observation, biochemical properties and other molecular feature gave confirmation of this laccase as “Yellow” laccase found in many *Pleurotus* spp.
